# Preparation and Optimization of Thermochromic Microcapsules as a Ternary System of Crystal Violet Lactone: Bisphenol A: Decanol Encapsulated with Urea Formaldehyde Resin in a UV-Curable Primer

**DOI:** 10.3390/polym17070851

**Published:** 2025-03-22

**Authors:** Yuming Zou, Xiaoxing Yan

**Affiliations:** 1 Co-Innovation Center of Efficient Processing and Utilization of Forest Resources, Nanjing Forestry University, Nanjing 210037, China; zou_yuming@njfu.edu.cn; 2 College of Furnishings and Industrial Design, Nanjing Forestry University, Nanjing 210037, China

**Keywords:** thermochromic microcapsule, urea formaldehyde resin, UV primer

## Abstract

Coatings can achieve the property of changing color with temperature variations by adding thermochromic microcapsules, which can bring a variable surface to the substrate. Ultraviolet ray (UV)-cured primers have the advantages of a fast curing rate, low-temperature curing, and low pollution. Thermochromic microcapsules can expand the application range of UV primers. Thermochromic microcapsules were synthesized through an orthogonal test, using crystal violet lactone, bisphenol A, and decanol as the core materials in a 1:4:50 mass ratio, with urea formaldehyde resin as the wall material. The effects of the addition of batches of the urea, the mass ratio of the formaldehyde solution to the urea, the hydrophilic–lipophilic balance (HLB) value of the emulsifier, and core-to-wall mass ratio on microcapsules yields, encapsulation rates, thermochromic color differences (ΔE), and formaldehyde releases during synthesis were investigated. The results were normalized, with the thermochromic ΔE as the primary reference for analysis. The results indicate that the HLB value of the emulsifier was the key factor that affected the microcapsule performance. In a single-factor test, the HLB value was adjusted within the range of 6.00 to 10.00. It was found that when the HLB value was 10.00, the microcapsules exhibited the best comprehensive performance, with a yield of 43.29%, an encapsulation rate of 45%, a thermochromic ΔE of 4.60, and a formaldehyde concentration released of 1.310 mg/L. The 11# microcapsules with the optimal morphology and better comprehensive performance were compared with the best 14# microcapsules. Different amounts of these microcapsules were added to the UV primer to investigate the effects of the 11# and 14# microcapsules on the mechanical and optical properties of the UV primer. The main component of the UV primer was polyurethane acrylic resin, propylene glycol diacrylate, and hexanediol diacrylate. When 14# microcapsules were added to the UV primer at a concentration of 10%, the primer exhibited the best comprehensive performance, with a fracture elongation of 17.44%, a roughness of 0.15 μm, and a visible light transmittance of 83%. Microcapsule technology was used to modify UV primers, endowing them with thermochromic properties and expanding the application range of thermochromic microcapsules.

## 1. Introduction

In recent years, research on color-changing materials has become increasingly popular. More and more products with color-changing materials have been adopted in people’s daily lives [1,2,3]. With the advancement of science and technology, a microcapsule field is gradually emerging. To meet the diversified needs of people, scientists are committed to developing a variety of functions of microcapsules [4,5,6,7]. Color-changing microcapsules are a new type of microcapsule that change color when subjected to the effects of the outside world, such as light, temperature, electricity, and force. [8,9,10]. With the development of smart homes and functional coatings, they are no longer limited to the traditional protective and decorative roles. More and more new coatings are beginning to have the ability to be intelligent and respond to changes in the external environment [11]. Wooden furniture faces various challenges in daily use [12,13,14]. In addition to strengthening the structural strength of wooden furniture, coatings can to some extent protect furniture from negative environmental factors [15,16]. Thermochromic coatings are capable of automatically changing color as the temperature changes and thus have a wide range of research and application prospects. Ultraviolet ray (UV)-curable coatings have been used in many fields due to their advantages of a fast curing speed, low-temperature curing, and low pollution [17,18,19]. At present, coatings are endowed with self-healing [20], antibacterial [21], flame-retardant properties [22], and more through various means [23,24]. However, existing UV coatings often lack color-changing properties, which limits their use in some special applications [25]. Therefore, the technology of incorporating thermochromic microcapsules into the UV coatings can not only impart thermochromic properties to the coatings and expand their applications but also improve the comprehensive performance and market competitiveness of the coatings to a certain extent [26].

Microcapsules, as wall–core particles, can provide unique functionality in various applications by encapsulating a core with a specific function through an outer wall [27,28]. There are also many modification methods in the field of color-changing coatings, such as 3D printing and structural color, which have been introduced to construct color-changing coatings [29,30,31]. While using various methods to modify the surface of furniture, attention should also be paid to maintaining the performance of the coating itself [32,33]. Among these methods, microcapsules have the characteristics of a low cost and simple preparation. Many fossil-based and biobased microcapsules have also long been widely used in various aspects of people’s daily lives [34,35]. Through microcapsule technology, coatings can achieve better properties, including thermochromic functions [36]. Over the past few decades, microencapsulation technology has gradually been widely used in the fields of coatings, pharmaceuticals, and food [37,38]. Especially in recent years, researchers have been exploring how to improve the performance of the UV coatings through microcapsule technology to increase the durability of coatings while ensuring environmental friendliness. Tözüm et al. [39] prepared poly(methyl methacrylate)/thermochromic system and a poly(methyl methacrylate-comethacrylic acid)/thermochromic system of microcapsules using the emulsion polymerization method. The thermochromic system consisted of crystal violet lactone as a leuco dye, bisphenol A as a color developer, and 1-tetradecanol as a solvent. Microcapsules with different core/shell ratios were synthesized to examine the effect of core/shell ratio on the properties of microcapsules. The color changed between dark blue and light blue depending upon the temperature change. As a common wall material, a urea formaldehyde resin is widely used in the preparation of microcapsules, which can form microcapsules with a thermochromic function by combining with thermochromic materials, providing a new technological way to modify the color-changing property of UV coatings [40,41]. This study aims to explore the application of thermochromic microcapsules in the UV coatings. According to our previous research, the use of tetradecanol in the system made it difficult for the microcapsules and coatings to undergo color changes in the room temperature range [42]. It has been shown that the use of decanol could cause the microcapsules to undergo a phase change at low temperatures, which would allow the system to undergo a color change [43]. The preparation process of the thermochromic microcapsules was optimized by orthogonal and single-factor tests using urea formaldehyde resin as the wall material and crystal violet lactone, bisphenol A and decanol as the core materials. The comprehensive performance of the thermochromic microcapsules and the optical and mechanical properties of the UV coatings were also analyzed under different conditions. The study showed that the preparation process of the thermochromic microcapsules and the addition rate of the thermochromic microcapsules had a significant effect on the properties of the UV coatings. The addition of microcapsules at the appropriate rate can give the coatings the thermochromic property and at the same time effectively maintain the comprehensive performance of the UV coatings. Thus, the application of thermochromic microcapsules in the field of smart home and functional coatings can be broadened. Through the preparation and optimization of the thermochromic microcapsules, this study provides a new idea for the research and development of smart coatings in the future and also provides technical support for the green development and sustainable innovation of coating industries. The coating preparation process can also further enhance the coating performance, so in future research, UV coatings can be applied to wood surfaces to explore the influence of preparation process on coating performance [44,45,46].

Thermochromic microcapsules were synthesized by orthogonal tests using crystal violet lactone, bisphenol A, and decanol as core materials and urea formaldehyde resin as a wall material. The effects of addition batches of urea, the weight ratio of formaldehyde solution to urea (W_F_:W_U_), and the hydrophilic–lipophilic balance (HLB) values of the emulsifier and core–wall mass ratios on microcapsules yields, encapsulation rates, thermochromic color difference (Δ*E*), and formaldehyde emissions during synthesis were investigated. The results showed that the HLB value of the emulsifier was the key factor affecting the microcapsule performance. A better preparation procedure for the thermochromic microcapsules was derived by setting the suitable HLB value of the emulsifier in a single-factor test. The better thermochromic microcapsules were added to the UV primer to manufacture a film, and the effect of the thermochromic microcapsules on the comprehensive performance of the UV primer was investigated.

## 2. Methods and Test Materials

### 2.1. Test Materials

The test materials are shown in Table 1. The UV primer used in the test was provided by Jiangsu Haitian Technology Co., Ltd., Zhenjiang, China. The main components of the UV primer included epoxy acrylic resin, polyester acrylic resin, trihydroxy methacrylate, TriMet acrylate, photo initiator, defoamer, leveling agent, etc. The solid content of the UV primer was more than 98.0%.

### 2.2. Synthesis Method of Thermochromic Microcapsules

Four factors, the addition batches of urea, the mass ratio of the formaldehyde solution to the urea (W_F_:W_U_), the HLB value of the emulsifier, and the core–wall mass ratios in the preparation of thermochromic microcapsules, were selected for orthogonal tests to determine the most influential factor among them. A single-factor test was then conducted with the most influential factor as the variable. The orthogonal test design is shown in Table 2, while Table 3, Table 4 and Table 5 show the details of the orthogonal tests, the materials of the orthogonal tests and the materials of single-factor tests, respectively. The calculation for the HLB values of Triton X-100 and gum Arabic powder for the tests of a reaction system is shown in Equation (1), and that of Span-80 and gum Arabic powder for the single-factor test is shown in Equation (2). *H* is the HLB value, *R*_1_ is the ratio of Triton X-100, *R*_2_ is the ratio of gum Arabic powder, and *R*_3_ is the ratio of Span-80.

*H* = *R*_1_ × 13.4 + *R*_2_ × 8.0 (1)

*H* = *R*_2_ × 8.0 + *R*_3_ × 4.3 (2)

In the methods for batch addition of urea, method A is a one-time addition; method B comprises a first addition of 55%, a second addition of 40%, and a final addition of 5%; and method C is a batch addition of 65%, 30%, and 5%.

Take microcapsule 14# for example:

Method of preparing and dispersing a thermochromic compound: The water bath (DF-101S, Shanghai Qiuzuo Scientific Instrument Co., Ltd., Shanghai, China) was adjusted to 50 °C, and 80 g of the decanol was weighed and put into a beaker, after which 4.80 g of bisphenol A and 1.60 g of crystal violet lactone were added using a mass ratio of crystal violet lactone: bisphenol A: decanol of 1:3:50. A stirrer was put into the mixture and stirred at 400 rpm for 1.5 h. After that, the thermochromic compound was cooled to room temperature. A 1.74 g measure of gum Arabic and 1.02 g of Triton X-100 were measured as emulsifiers. A total of 52.52 mL of distilled water was added, and 4.15 g of thermochromic compound was added. The system was heated up to 65 °C and stirred at the corresponding higher speed for 30 min. The stirred solution was placed in an ultrasonic emulsifier and subjected to ultrasonication for 5 min to ensure that the emulsifiers were uniformly encapsulated on the outer surface of the core materials.

Method of preparing the wall material: The first batch of urea and 8.42 g of formaldehyde were weighed according to Table 5, and 125.00 mL of distilled water was added to the mixture, which was then placed in a water bath and stirred at room temperature until dissolved. Triethanolamine was added to adjust the pH of the solution to 8.5; then, the beaker was sealed with the water bath heated to 70 °C and stirred at 300 rpm for 1 h.

Method of preparing the thermochromic microcapsules: The core material emulsion after ultrasonication was placed in a water bath at 35 °C after standing at room temperature with slow stirring. The wall material solution and the second batch of urea were added to the core material solution, and then the rotational speed of the water bath was adjusted to 500 rpm. Then, 0.58 g of SiO_2_ and 0.58 g of NaCl were added, 8% citric acid monohydrate was added drop by drop, and the pH of the solution was adjusted to 2.5. Then, the reaction was carried out for 1 h. After the reaction was completed, the third batch of urea was added. The temperature was raised to 68 °C, and the rotational speed was reduced to 250 rpm. The reaction was continued for 30 min to obtain the solution of thermochromic microcapsules. The prepared product was sealed in the beaker and left to stand for three days and then filtered by a vacuum pump using the distilled water, and the light blue powder obtained after drying in an oven at 35 °C was the thermochromic microcapsules.

### 2.3. The Synthesis Method of Thermochromic UV Primer

The size of the mold for film preparation of the UV primer was 50 mm × 50 mm × 20 mm, and the material table used for mixing UV primers is shown in Table 6.

Method of preparing the UV primer for the orthogonal test and single-factor test: The thermochromic microcapsules to be tested were added to the UV primer with the addition of 0.300 g, keeping the total mass of microcapsules and UV primer as 1.000 g. After mixing uniformly, the mixture was poured into the mold. The mixed primer was naturally leveled, and then the mold was put onto a conveyor belt of a single-lamp curing machine (620#, Huzhou Tongxu Machinery Co., Ltd., Huzhou, China). The transfer speed was adjusted to 0.01 m/s. The curing time was about 1 min, and the cured UV primer was taken off the mold for the color-changing property test.

Method of preparing the UV primer for the coating test: The best microcapsules from the single-factor test were added to the UV primer at 0%, 5%, 10%, 15%, 20%, and 25%, keeping the total mass of the thermochromic microcapsules and the UV primer as 1.000 g. After mixing, the mixture was evenly poured into the mold for the preparation of the coating film, or kept at a total mass of 0.500 g, to be coated onto a glass plate. After the mixed primer was naturally leveled, the mold was placed on the conveyor belt of the single-lamp curing machine, the transfer speed was adjusted to 0.01 m/s, and the curing time was about 60 s. The cured UV primer was demolded and tested for optical and mechanical properties, in which the UV primer without the added microcapsules was the blank control group.

### 2.4. Test and Characterization

#### 2.4.1. Yield and Encapsulation Rate Test

The yield is denoted as *P*, the mass of the prepared microcapsules is denoted as *m_p_*, and the masses of the components of the wall and core materials used are denoted as *m_r_*. The yield was calculated as in Equation (3).

*P* = (*m_p_*/*m_r_*) × 100% (3)

The encapsulation rate is denoted as *P_c_*. The microcapsules were weighed with the mass of *m*_1_, fully ground, added to a beaker, immersed in anhydrous ethanol, and heated in an oven at 60 °C for 2 h. The mixture was stirred well with a glass rod every 15 min. After the reaction was completed, the microcapsules were rinsed and filtered with anhydrous ethanol. The filtered microcapsules were placed in the oven at 60 °C for drying. The mass of the residue was weighed and noted as *m*_1_. The encapsulation rate was calculated as in Equation (4).

*P_c_* = [(*m*_1_ − *m*_2_)/*m*_1_] × 100% (4)

#### 2.4.2. Color Changing Property and Formaldehyde Emission Test

Color changing property: The color difference (Δ*E*) of the microcapsule powder could not be directly obtained because of the limitation of the precision of the test device, so the microcapsules were added into the UV primer at a fixed mass ratio of 30%. The total mass was controlled to be kept the same. In this way, under controlled conditions, the Δ*E* between different microcapsules can be effectively reflected and measured. The prepared coating film was put into the refrigerator and frozen for some time to ensure that the microcapsules cooled down sufficiently. A portable color difference meter (SEGT-J, Beijing Times Mountain Technology Co., Ltd., Beijing, China) was used to measure and record the *L*, *a* and *b* values of the UV primer. The temperature values taken depend on the purpose and the conditions of the test. The microcapsules prepared in this test were low-temperature color-changing microcapsules. Microcapsules are expected to be used for the preparation and practical application of UV coatings for low-temperature color change on wood substrate surfaces. Unlike most other tests, the lowest temperature of the test refrigerator, −20 °C, was taken as the minimum temperature because, after approaching and passing 60 °C, the microcapsules’ color hardly changed, and they were maintained at a light blue color. Compared to the blue color of the low temperature, as it warmed up to light blue, there was no reference value, so 60 °C was chosen as the maximum temperature. The color difference was calculated as the average of the 4 tests of the color difference values at the two temperatures of −20 °C and 60 °C. Because UV primers did not originally have thermochromic changing properties, the test results can be recorded as the data of the color changing property of microcapsules.

According to GB/T 11186.3-1989 [47], the *L* value represented a brightness value of the sample, the *a* value represents a red-green value of the sample, and the *b* value represents a yellow-blue value of the sample. The test data of the UV primer at high temperature were *L*_1_, *a*_1_, *b*_1_; the test data of the film at low temperature were *L*_2_, *a*_2_, *b*_2_; and the color difference Δ*E* was calculated according to Equation (5), in which Δ*L* = *L*_2_ − *L*_1_, Δ*a* = *a*_2_ − *a*_1_, Δ*b* = *b*_2_ − *b*_1_.

Δ*E* = [(Δ*L*)^2^ + (Δ*a*)^2^ + (Δ*b*^2^]^1/2^ (5)

Formaldehyde emission: During the preparing process of the thermochromic microcapsules, after the beaker was set for 3 days, the formaldehyde emission from the system in the beaker was detected using an air quality detector (DM105D, Shenzhen Langdeli Technology Co., Ltd., Shenzhen, China). The range of the formaldehyde emission of the device was 0–1.999 mg/m^3^.

#### 2.4.3. Normalized Analysis

The data of yield, encapsulation rate, ΔE, and formaldehyde emission of the thermochromic microcapsules were analyzed by equal-weight normalization. The data results of orthogonal and single-factor tests of the sample microcapsules were quantitatively evaluated and were used to determine the maximum influential factors on the comprehensive performance of the microcapsules. At the same time, the selection of the better microcapsules could be determined to a certain extent. The results of the test data were converted into positive results using the great value method, in accordance with the calculation of Equation (6), in which each test datapoint *n_x_* is divided by the maximum value of the corresponding datapoint *n_m_* in the results of this item and multiplied by 100 to obtain the corresponding score of 0–100. Then, the sum of the scores is divided by the total number of items N to obtain the average value of the equal-weight score of the sample, *n*, which embodies the comprehensive performance of the sample.

*n* = [Σ(*n_x_*/*n_m_*)/N] × 100 (6)

Great value processing method of formaldehyde emission: The maximum range of 1.999 mg/m^3^ of the device was used as the standard as the great value. The formaldehyde emission was subtracted from that obtained, thus obtaining the positive formaldehyde emission processing data.

#### 2.4.4. Microcosmographic Analysis

Optical microscope (OM): An appropriate amount of the sample was placed on a slide and placed under an optical microscope (AX10, Carl Zeiss AG, Jena, Germany), which was adjusted to the appropriate magnification for observation.

Scanning Electron Microscope (SEM) (Quanta-200, Thermo Fisher Scientific, Waltham, MA, USA): The appropriate amount of the sample was dried and taken for gold spraying. The sample was placed inside the scanning microscope, which was then adjusted to the appropriate magnification to observe after the air pressure met the conditions.

#### 2.4.5. Chemical Composition Test

The chemical composition of the prepared thermochromic microcapsules and the UV primers was analyzed by infrared spectroscopy (VERTEX 80V, Bruker, Germany).

#### 2.4.6. Optical Properties Test

Glossiness: According to GB/T 4893.6-2013 [48], the glossiness of the UV primer was tested by using a gloss meter (DT60, Changzhou Duder Precision Instrument, Co., Ltd., Changzhou, China), the glossiness of the UV primer was tested and recorded at three angles of incidence (20°, 60°, and 85°), and the differences were compared [49].

Transmittance: A UV spectrophotometer (U-3900, Hitachi Instruments Co., Ltd., Suzhou, China) was used to test the transmittance of the UV primer in the visible wavelength range. Transmittance is the ratio between the actual light intensity and the original light intensity when a beam of light passes through the UV primer [50,51].

#### 2.4.7. Mechanical Property Test

Elongation at break: The UV primer was made into the standard specification and then stretched at a speed of 0.5 mm/min by clamping the ends of the film with the clamping arms of a universal mechanical testing machine (AG-IC10OKN, Shimadzu, Kyoto, Japan) until it was broken. The elongation at break of the UV primer was calculated as shown in Equation (7), where *e* denotes the elongation at break of the UV primer at the point of break, *L*_0_ is the initial distance between the upper and lower clamping arms when the UV primer is stretched, and *L*′ is the distance between the upper and lower clamping arms when the UV primer is broken.

*e* = [(*L*′ − *L*_0_)/*L*_0_] × 100% (7)

Roughness: A roughness meter (J8-4C, Shanghai Taeming Optical Instrument Co., Ltd., Shanghai, China) was used to measure and record the values to derive the trend of roughness between different UV primers [52].

## 3. Results and Discussion

### 3.1. Results Analysis of the Preparation of the Thermochromic Microcapsules

#### 3.1.1. Analysis of Yield and Encapsulation Rate of Thermochromic Microcapsules Prepared by Orthogonal Test

Table 7 shows the analysis of the yield results of the thermochromic microcapsules prepared by the orthogonal test. The highest yield of 44.54% was recorded for sample 6#, followed by a 43.03% yield for sample 3# and a 41.26% yield for sample 9#. An extreme deviation indicated that the addition of batches of urea had the greatest effect on the yield of the thermochromic microcapsules. For the yield of the thermochromic microcapsules, the four factors had a primary level of A > D > B > C and the recommended preparation process was A1B3C1D1. The yield of microcapsules with a single addition batch of urea was significantly higher than that of the other two types because the single addition of urea resulted in a more complete reaction and higher material utilization throughout the process.

Table 8 shows the analysis table of results of encapsulation rate of the thermochromic microcapsules obtained from orthogonal tests. Sample 1# had the highest encapsulation rate of 71.0%, followed by sample 2# with a 57.0% encapsulation rate and sample 7# with a 50.0% encapsulation rate. The extreme deviation analysis showed that W_F_:W_U_ and emulsifier HLB value had the most significant effect on the thermochromic microcapsules’ encapsulation rate. The order of precedence of the four factors was B = C > D > A, and the recommended combination of preparation processes was A1B1C1D1. As the W_F_:W_U_ decreased, the difficulty of forming the wall increased and the encapsulation rate decreased, although the free formaldehyde might have decreased. The HLB values also showed a huge effect in the analysis of the encapsulation rate results, but because the effect of core emulsification on the test was manyfold, it would be prioritized for further analysis in a single-factor test. As the HLB values increased, the bonding between core materials and wall materials might have deteriorated, resulting in a decrease in the encapsulation rate.

#### 3.1.2. Analysis of Color-Changing Property and Formaldehyde Emission of the Thermochromic Microcapsules by Orthogonal Test

In Table 9, the changes in Δ*E* and formaldehyde emission of thermochromic microcapsules are analyzed under different temperature conditions. The color of thermochromic microcapsules changed gradually from blue to colorless as the temperature increased from low to high. According to the Δ*E* value, the color difference value generally increased after the temperature was increased, indicating that the thermochromic microcapsules had the color-changing property. Sample 8# had the largest Δ*E* value of 3.500, which was the best performance. The next sample was 6#, with a Δ*E* value of 2.559, followed by 1#, with a Δ*E* value of 2.329. For formaldehyde emission, 6# and 5# had the smallest formaldehyde emission at 1.299 mg/m^3^ and 1.364 mg/m^3^.

Table 10 demonstrates the results analysis of Δ*E* of the thermochromic microcapsules in orthogonal tests. The results of extreme deviation analysis show that the emulsifier HLB values had the most significant effect on the color changing of thermochromic microcapsules. The order of influence of the four factors was C > A > D > B, and the recommended process combination was A3B3C1D3. Table 11 demonstrates the results of the analysis of formaldehyde emission in the orthogonal tests. The data were pre-processed at a maximum range for 1.999 mg/m^3^ of the device. The extreme deviation analysis showed that W_F_:W_U_ had the most significant effect on formaldehyde emission. The main order of the four factors was B > A > D > C, and the best recommended process combination was A2B2C1D2. Although the ΔE decreased and then increased as the HLB values increased, the other properties reflected better levels when the HLB values were 8 and 10. Thus, there was also a need for further exploration of the HLB values below 8. Combining the data of the urea batch and the W_F_:W_U_, the result of formaldehyde emissions showed that less formaldehyde addition would cause less emission.

#### 3.1.3. Normalization of Orthogonal Test Results and Comprehensive Performance Analysis of the Thermochromic Microcapsules of the Single-Factor Test

The negative factor of formaldehyde emission was converted to the value of the spread between it and the maximum value of 1.999 mg/m^3^, to obtain a positive factor that can be easily compared and assessed in a uniform manner with other positive factors. In this way, the further the value of formaldehyde emission was from 1.999 mg/m^3^, the larger the spread was, which indicated that its impact on the environment was smaller and its performance was better. Subsequently, the results of the test data were normalized and analyzed, and the indicators such as yield, encapsulation rate, Δ*E*, and formaldehyde emission were converted to standardized percentages uniformly through the great value method so that a comprehensive assessment could be carried out at the same scale. The final composite scores after normalization are shown in Table 12, with Sample 6# scoring the highest at 84.827 points, with the best performance, followed closely by Sample 1#, with a score of 82.954 points, with a high overall performance; Sample 8# ranked third, with a score of 80.059 points, with an overall performance that was also relatively good. This analysis not only demonstrated the comprehensive performance of each sample on various indicators but also provided quantitative data support for the subsequent analysis of the results.

Table 13 demonstrates the analysis of the results of the orthogonal test for the mean of the average of equal-weighted total scores. The extreme deviation analysis showed that the emulsifier HLB value had the most significant effect on the scores. The primary order of the four factors was C > B > D > A. In summary, the optimal process combination was A1B3C1D2, i.e., the process where the urea was added at one time, then W_F_:W_U_ was 1:0.8, the core–wall ratio was 1:1.2, and the emulsifier HLB value was 8. The main demanded property of the thermochromic microcapsules is the color-changing property, and the biggest factor influencing the color-changing property was also the emulsifier HLB value. Therefore, on this basis, the single-factor test was designed to follow the process, and the range of the emulsifier HLB value was set between 6 and 10 for testing to further validate the effect of emulsifier HLB value on the performance of thermochromic microcapsules.

Table 14 and Table 15 demonstrate the comprehensive performance results of the single-factor test microcapsules and the normalized scores of the sample results of the single-factor test. In the single-factor test, the HLB values of 10#–14# were 6, 7, 8, 9, and 10, respectively. Sample 14# was outstanding in all indicators; in particular, it had the best Δ*E* of 4.600 and the lowest formaldehyde emission of 1.310, and its total score was 96.285, which was significantly higher than the other samples. Therefore, sample 14# was selected as the best thermochromic microcapsules. Sample 11#, with an overall rating of 86.290, also performed well, ranking second in the normalized score; in particular, it had the highest yield of 49.58. The Δ*E* ranked second at 3.138, and its overall performance remained outstanding. The optimal preparation process was the one-time addition of urea, a W_F_:W_U_ of 1:0.8, a core–wall ratio of 1:1.2, and an emulsifier HLB value of 10.

The thermochromic microcapsule yield and encapsulation rate increased and then decreased as the HLB value increased, indicating that the closer the HLB value was to neutral, the better the microcapsules were formed and the higher the material utilization. As the emulsifier HLB value increased, the higher the thermochromic microcapsules Δ*E*, which indicated that for the core material, the high emulsifier environment better maintained its color-changing property. The formaldehyde emission was only higher when the HLB value was 6. This is because the combination of thermochromic microcapsule wall material and core material was incomplete when the HLB value was 6, and the formaldehyde in the wall material could not be well utilized.

#### 3.1.4. Analysis of the Microscopic Morphology of the Thermochromic Microcapsules

**Figure 1:**
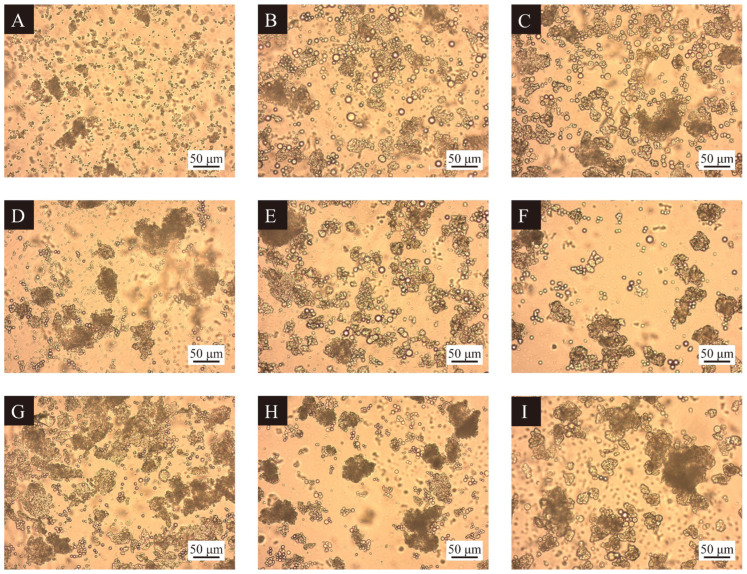
OM of microcapsules of orthogonal tests, (**A**–**I**) microcapsules 1#–9#.

As shown in Figure 1, the morphology of the thermochromic microcapsules did not change much with the increase in the addition of batches of urea. With the decrease in W_F_:W_U_, the agglomeration phenomenon of thermochromic microcapsules was reduced, and the particle size of the thermochromic microcapsules was increased. This is because the lower the W_F_:W_U_, the more dihydroxymethylurea was generated. The higher the cross-linking degree of microcapsules, the denser the wall material. The agglomeration of thermochromic microcapsules was serious when the emulsifier HLB value was 8. The morphology of microcapsules of 2#, 4#, and 9# was better when the emulsifier was 10. This indicates that the synthesis of the thermochromic microcapsules was suitable to be carried out in a hydrophilic and lipophilic environment close to neutrality. Meanwhile, as the core–wall ratio decreased, the particle size of thermochromic microcapsules became larger and the morphology became better while the agglomeration phenomenon became more serious. This is because, with the increase in wall material, thermochromic microcapsules are easier to shape, but there will be cross-linking with each other, leading to agglomeration.

**Figure 2:**
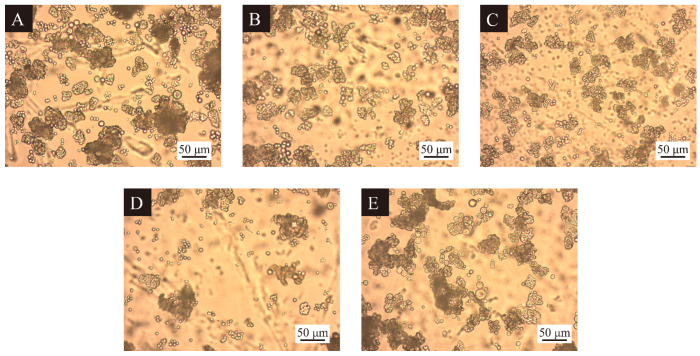
OM of microcapsules of single-factor tests, (**A**–**E**) microcapsules 10#–14#.

As shown in the OM of the microcapsule samples prepared in the single-factor test in Figure 2, the agglomeration phenomenon of the thermochromic microcapsules decreased and then increased as the HLB value of the emulsifiers continued to increase. The particle sizes of microcapsules 11#, 12#, and 14# were more uniform, corresponding to emulsifier HLB values of 7, 8, and 10, respectively, indicating that emulsifiers that were too lipophilic or hydrophilic would lead to the agglomeration phenomenon, which was not conducive to the synthesis and dispersion of the thermochromic microcapsules. At an emulsifier HLB value of 10, the thermochromic microcapsule 14# had the best morphology, less agglomeration phenomena, and a uniform particle size. Therefore, the neutral emulsifier HLB value was more suitable for the preparation of thermochromic microcapsules.

**Figure 3:**
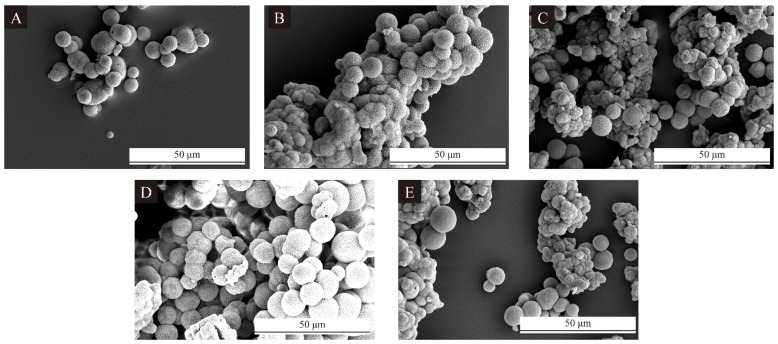
SEM of microcapsules of orthogonal test, (**A**–**E**) microcapsules 10#–14#.

**Figure 4:**
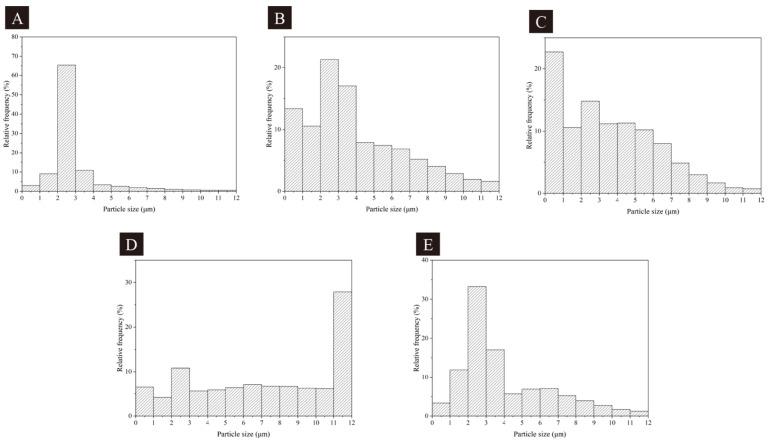
Particle size distribution of microcapsules for single-factor test, (**A**–**E**): microcapsules 10#–14#.

Combined with the single-factor microcapsule SEM graph in Figure 3 and the microcapsule particle size distribution in Figure 4, it can be found that the cross-linking phenomenon for microcapsule 10# was heavier and the particle size was uniform, but the microcapsule morphology was poorer. The particle size distribution for microcapsule 12# was uneven, distributed in 0–9 μm, which would significantly affect the microcapsule and the performance of the coating. Although the distribution of the particle size of microcapsule 13# was centralized, the microcapsules were too large, and the particle size was concentrated in more than 11 μm, which would affect the overall performance of the coating when added into the UV primer. Microcapsule 11# had the best morphology, with a concentrated distribution in 0–5 μm. Microcapsule 14# had some cross-linking between the microcapsules, but the particle size was more uniform. The distribution of the particle size was concentrated between 1 and 4 μm. It can be found that the morphology of microcapsule 11# was the best, and there was some cross-linking for microcapsule 14#, but the particle sizes of both microcapsules were more uniform. With the increase in the HLB values, the microcapsule microcosmography did not change much. But the range of the microcapsule particle size distribution first increased and then decreased, which represents a more uniform particle size distribution of microcapsules prepared under the condition of biased neutral emulsifier HLB value.

#### 3.1.5. Analysis of Chemical Composition of the Thermochromic Microcapsules

**Figure 5:**
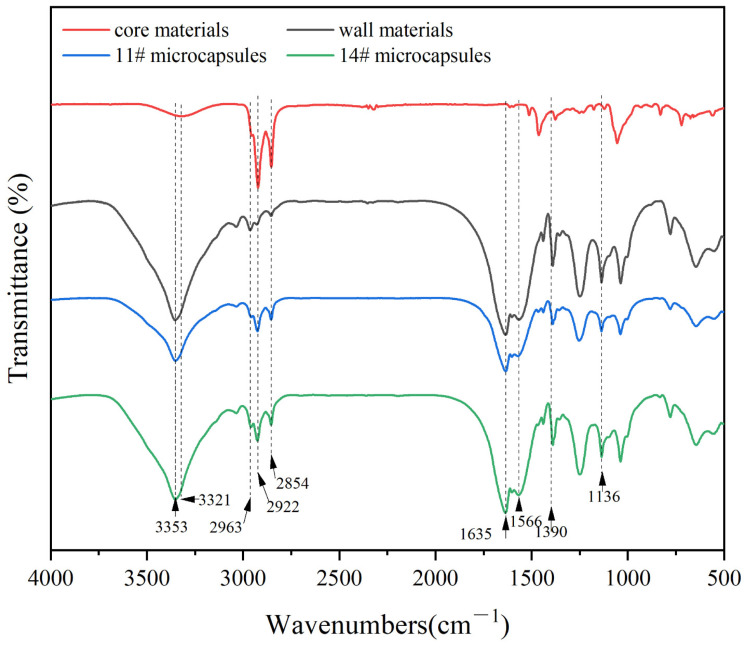
Infrared spectroscopy of core materials, wall materials, and microcapsules.

From Table 16 and Figure 5, 3321 cm^−1^ and 2922 cm^−1^ are the telescopic vibration peaks of -OH conjugation and the telescopic vibration peak of C-CH_3_, respectively. Furthermore, 1513 cm^−1^ and 1463 cm^−1^ are the telescopic vibration peaks of the benzene ring C=C, and 1251 cm^−1^ and 1055 cm^−1^ are the absorption peak of C-OH and the telescopic vibration peak of C-O, respectively. These are the characteristic peaks of bisphenol A. The telescopic vibration peak of carbonyl group C=O of the lactone ring appeared at 1613 cm^−1^, and the symmetrical telescopic vibration peaks of the ester group C-O-C appeared at 1177 cm^−1^ and 1055 cm^−1^, which proves that the lactone ring was in a closed state at this time. The crystal violet lactone only partially reacted with bisphenol A, opening the ring to form a conjugated color-emitting system. The 3321 cm^−1^ appeared as the -OH-absorption peak in the carboxyl group, indicating that the lactone ring was broken, the conjugated system was destroyed, the structure was changed, and the color changed [42].

The stretching vibration peak of N-H bond and O-H bond superposition appeared at 3353 cm^−1^. The asymmetric stretching vibration peak of -CH_2_-, the carbonyl stretching vibration peak of the secondary acyl group, and the CH_3_O characteristic peak appeared at 2963 cm^−1^, 1635 cm^−1^ and 1136 cm^−1^, respectively. The amide N-H bond bending vibration and C-N bond stretching vibration peaks appeared at 1566 cm^−1^. These peaks indicate the successful preparation of urea formaldehyde resin.

The ester carbonyl C=O absorption peaks of the non-lactone ring structure appeared at 1635 cm^−1^, and the symmetric telescopic absorption peaks at 1390 cm^−1^ correspond to the carboxylate salt, which proves that the lactone ring in the molecule opens up and forms a conjugated chromogenic structure. The 2922 cm^−1^ peak is attributed to the telescopic vibrational peaks of the C-CH_3_ in the decanol and the crystal violet lactone. These characteristic peaks indicate the successful preparation of thermochromic microcapsules.

Combined with the test results of the other properties of microcapsules, microcapsule 14# had the best overall performance when the HLB value was 10.00, with a yield of 43.29%, an encapsulation rate of 45%, a thermochromic Δ*E* of 4.60, and a formaldehyde release concentration of 1.310 mg/m^3^. The microcapsule 11#, which had the best morphology and a good overall performance, was selected as a comparison with the best microcapsule 14#. These two microcapsules were added to the UV primer, and the various properties of the UV primer were tested comparatively to determine the optimal addition rate of the thermochromic microcapsules in the UV primer.

Initially, crystal violet lactone presented a closed-ring structure, which played the role of an electron-delivering compound in the color-changing system, while bisphenol A existed as an electron-absorbing compound. At low temperatures, the -OH molecule in bisphenol A reacted with the central carbon atom of the crystal violet lactone, resulting in the rupture of the crystal violet lactone ring, leading to the formation of a double-bonded structure with a conjugated structure, which ultimately resulted in a blue coloration of the core. At elevated temperatures, the conjugated double bond broke due to the melting action of the solvent, causing the crystal violet lactone to reform the lactone ring structure, resulting in a colorless appearance of the core.

### 3.2. UV Primer Performance Analysis

#### 3.2.1. Analysis of the UV Primer’s Optical Properties

Table 17 shows the effects of different additions rates from 0% to 25% of thermochromic microcapsules 11# and 14# on the glossiness and transmittance of the UV primer. The glossiness changes of the UV primer at a 60° incidence angle were analyzed with different addition rates of microcapsules. With the increase in the addition rates of microcapsules, the glossiness of the UV primer with thermochromic microcapsule 11# gradually decreased, and then slightly rebounded at 25%. Microcapsule 11# had better microscopic morphology but made the flatness of the UV primer rebound at high addition rates. The glossiness of the UV primer with thermochromic microcapsule 14# first increased and then decreased. The rate of losing glossiness first increased and then decreased. When the addition rate reached 20% and above, the glossiness showed a sharp decline. This is mainly due to the higher rate of thermochromic microcapsules, which made the UV primer not level well and become rougher. The flatness was affected after curing, which in turn reduced the ability of light reflection, leading to a reduction in glossiness. With microcapsule 11# at an addition rate of 5%, the glossiness of the UV primer was optimal for the 43.7 GU, in 15% down to 35.8 GU. With microcapsule 14# at the same rate of 15%, the glossiness remained at a high level of 88.3 GU. At the same time, because microcapsule 14# agglomeration was more serious compared with that of 11#, with an amount of 10% or less, the surface gloss of the UV primers with microcapsule 14# was more affected by the microcapsule agglomeration, there were local differences in the leveling, and there was a big difference in the glossiness of the UV primer at each position.

**Figure 6:**
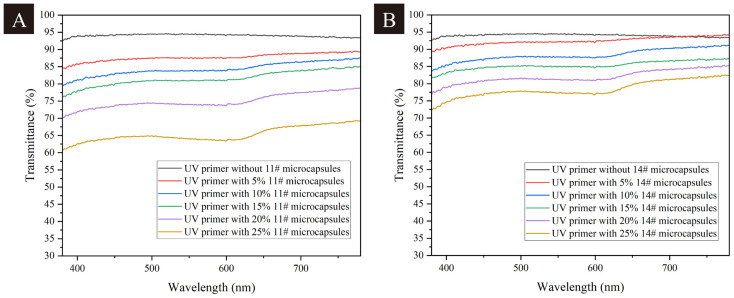
(**A**) UV visible transmittance of UV primer with and without microcapsule 11#; (**B**) UV visible transmittance of UV primer with and without microcapsule 14#.

As shown in Figure 6, the UV-visible light transmittance decreased with the increase in the addition rate of the thermochromic microcapsules. The transmittance of the UV primer without adding microcapsules was 94.06%. With the increase in the addition rates, the transmittance decreased gradually and finally decreased to 65.31% when the addition rate of microcapsule 11# was 25% and decreased to 78.41% when the addition rate of microcapsule 14# was 25%. The addition of thermochromic microcapsules, especially when their addition rate was too high, increased the shading property of the UV primer, resulting in a significant decrease in its ability to transmit visible light, because the color of microcapsules hinders the transmittance of light. Considering the effect of the addition rate of thermochromic microcapsules on glossiness and light transmittance, microcapsule 14# with an addition of 10–15% was more in line with the requirements in terms of optical properties.

#### 3.2.2. Analysis of UV Primer Mechanical Properties

As shown in Table 18, showing the effects of the addition of 11# and 14# microcapsules at different addition rates on the elongation at break and roughness of the UV primer, the addition of thermochromic microcapsules 11# and 14# in the UV primer had a significant effect on the elongation at break and roughness of the UV primer. For thermochromic microcapsule 11#, the elongation at break was significantly increased at addition rates from 5% to 10%, reaching a maximum value of 15.3% at 10%, but it decreased sharply beyond 15%. In contrast, thermochromic microcapsule 14# maintained a higher toughening effect in the 5–15% addition range, the change in roughness was smoother, and the surface quality was more uniform. Considering these points together, thermochromic microcapsule 14# showed the best mechanical properties at 10% addition, with excellent results in elongation at break and roughness. Therefore, synthesizing the optical properties of the UV primer, the optimal thermochromic microcapsule addition amount is 10% for microcapsule 14# in the UV primer to obtain the best comprehensive performance of the UV primer.

#### 3.2.3. Analysis of UV Primer Morphology

**Figure 7:**
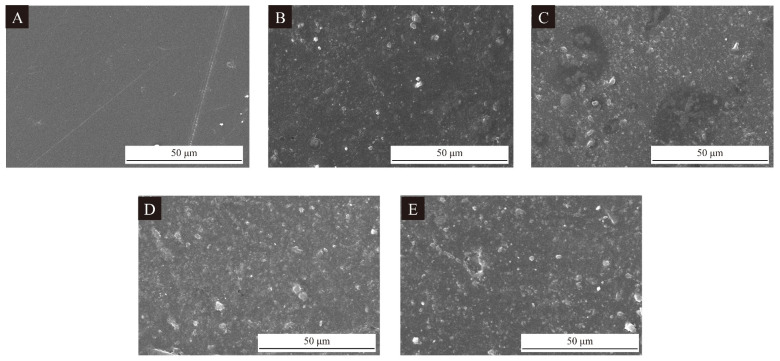
SEM of the UV primer, (**A**) the UV primer without microcapsules, (**B**) the UV primer with 5% of microcapsule 14#, (**C**) UV primer with 10% of microcapsule 14#, (**D**) UV primer with 15% of microcapsule 14#, and (**E**) UV primer with 15% of microcapsule 11#.

Figure 7 shows the SEM of UV primers with the addition of thermochromic microcapsules 11# and 14# at different rates. The surface of the UV primer without thermochromic microcapsules was very smooth, and with the increase in the addition rate of microcapsules, the surface of the UV primer gradually became rough, and the unevenness of the UV primer due to the agglomeration of the microcapsules could be observed by comparing the optimal UV primer with 15% of microcapsule 14# and the one with 15% microcapsule 11#. The surface SEM of the UV primer with 15% of microcapsule 11# was even worse, with larger pits and more agglomerations. Considering that the addition rate directly affected the Δ*E*, the optimal surface morphology with 10% thermochromic microcapsule 14# was acceptable.

#### 3.2.4. Analysis of the Chemical Composition of UV Primers

**Figure 8:**
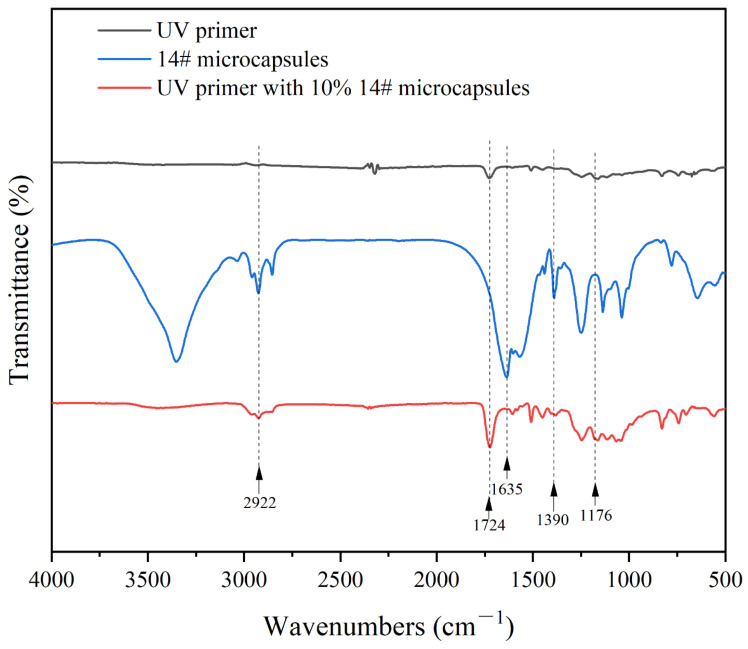
Infrared spectra of the UV primer, microcapsule 14# and UV primer with 10% of microcapsule 14#.

Figure 8 shows the infrared spectra of the UV primers and the thermochromic microcapsules. The C-O and C=O stretching vibration peaks were 1176 cm^−1^ and 1724 cm^−1^, respectively, which were the characteristic peaks belonging to the polyester acrylic resins, trihydroxymeth acrylates, and trimethyl acrylates, which were the main components of the UV primer. The C-CH_3_ stretching vibration peaks of bisphenol A, the main component of the core material, and epoxy acrylic resin, the main component of the UV primer, were observed at 2922 cm^−1^, which was stronger in the absorption curve of the UV primer with 10% of microcapsule 14#. The 1635 cm^−1^ showed an ester carbonyl C=O absorption peak with a non-lactone ring structure, and the 1390 cm^−1^ corresponded to the symmetrical absorption peak of carboxylates, proving that the lactone ring in the molecule opened and formed a conjugated color-emitting structure.

This shows that the addition of thermochromic microcapsules has no effect on the UV primer curing and film-forming reaction process and proves that the thermochromic microcapsules could stably exist in the UV primer. When thermochromic microcapsule 14# was added to the UV primer at an addition rate of 10%, the UV primer exhibited the best overall performance with the UV visible light transmittance of 88.33%, a 60° glossiness of 80.3 GU, a rate of glossiness loss of 28.37%, an elongation at break of 17.4%, and a roughness of 0.153 μm.

## 4. Conclusions

Thermochromic microcapsules with urea formaldehyde resin as the wall material and crystal violet lactone: bisphenol A: decanol as core material were prepared and optimized by orthogonal and single-factor tests. The effects of the addition batches of urea, the W_F_:W_U_, the HLB value of the emulsifier, and the core–wall ratio on the comprehensive performance of the thermochromic microcapsules were investigated. It was concluded that the largest factor influencing the comprehensive performance of thermochromic microcapsules was the HLB value of the emulsifier. The better preparation process of the thermochromic microcapsules was as follows: one-time urea addition, W_F_:W_U_ of 1:0.8, core–wall ratio of 1:1.2, and HLB value of 10. Microcapsule 14# had the best overall performance when the HLB value was 10.00, with a yield of 43.29%, an encapsulation rate of 45%, a thermochromic Δ*E* of 4.60, and a formaldehyde emission of 1.310 mg/m^3^, and the total score was 96.285. The results show that 10.0% addition of thermochromic microcapsule 14# reduced the glossiness of the UV primer, which rebounded at 25% of the added rate and weakened the transmittance of the UV primer. The elongation at break of the UV primer showed a tendency of increasing and then decreasing, and the roughness firstly decreased and then gradually increased. The comprehensive performance of the UV primer was better when the addition rate of thermochromic microcapsule 14# was 10%, the UV visible light transmittance was 88.33%, the glossiness was a 60° glossiness of 80.3 GU, the rate of glossiness loss of 28.37%, the elongation at break was 17.4%, and the roughness was 0.153 μm. In the future, the exact temperature of color-changing UV primers with thermochromic microcapsules will be determined, and the thermochromic UV primer will be applied to a substrate such as wood for further practical application. As important parts of the performance of thermochromic microcapsules, further testing of thermal stability and leakage in the future can further reflect the usability of microcapsules.

## Figures and Tables

**Table 1:** Test materials.

Test Materials	Molecular Formula	Molecular Mass	Purity	Manufacturer
Crystal violet lactone	C_26_H_29_N_3_O_2_	415.527	AR	Wuhan Huaxiang Biotechnology Co., Ltd., Wuhan, China
Bisphenol A	C_15_H_16_O_2_	228.286	AR	Shanghai Haiyu Chemical Co., Ltd., Shanghai, China
Decanol	C_10_H_22_O	158.28	AR	Shanghai McLean Biochemical Technology Co., Ltd., Shanghai, China
Urea	CH_4_N_2_O	60.06	AR	Guangzhou Suixin Chemical Co., Ltd., Guangzhou, China
Formaldehyde solution	CH_2_O	30.03	37%	Shandong Xinjucheng Chemical Technology Co., Ltd., Jinan, China
Triethanolamine	C_6_H_15_NO_3_	149.19	AR	Shandong Chengkai New Material Co., Ltd., Linyi, China
Citric acid monohydrate	C_6_H_10_O_8_	210.139	AR	Jinan Xiaotian Chemical Co., Ltd., Jinan, China
Gum Arabic powder	N/A	-	AR	Nanjing Jinyou Biotechnology Co., Ltd., Nanjing, China
Triton X-100	C_16_H_26_O_2_	250.376	AR	Shandong Yusuo Chemical Technology Co., Ltd., Linyi, China
Span-80	C_24_H_44_O_6_	428.6	AR	Shandong Yusuo Chemical Technology Co., Ltd., Linyi, China

**Table 2:** Design of orthogonal tests.

Levels	Factor A Urea Batch	Factor B W_F_:W_U_	Factor C HLB Values	Factor D Core–Wall Ratio
1	A	1.6:1	8.00	1:1
2	B	1.2:1	10.00	1:1.5
3	C	0.8:1	13.40	1:2

**Table 3:** Detail of orthogonal test.

Sample (#)	Factor A	Factor B	Factor C	Factor D
1	A	1.6:1	8.00	1:1
2	A	1.2:1	10.00	1:1.5
3	A	0.8:1	13.40	1:2
4	B	1.6:1	10.00	1:2
5	B	1.2:1	13.40	1:1
6	B	0.8:1	8.00	1:1.5
7	C	1.6:1	13.40	1:1.5
8	C	1.2:1	8.00	1:2
9	C	0.8:1	10.00	1:1

**Table 4:** Materials required in orthogonal test.

Sample (#)	Urea Batch	Urea (g)	Formaldehyde (g)	Distilled Water for Wall Material (mL)	Gum Arabic (g)	Triton X-100 (g)	Distilled Water for Core Material (mL)	Core Materials (g)	NaCl (g)	SiO_2_ (g)
1	A	8.00	16.84	288.00	9.60	-	182.40	14.40	2.02	2.02
2	A	8.00	12.62	256.00	3.58	2.11	108.09	8.53	1.19	1.19
3	A	8.00	8.42	125.00	-	2.07	39.39	3.11	0.44	0.44
4	B	8.00	16.84	288.00	3.02	1.78	91.20	7.20	1.01	1.01
5	B	8.00	12.62	256.00	-	8.53	162.13	12.80	1.79	1.79
6	B	8.00	8.42	125.00	2.76	-	52.52	4.15	0.58	0.58
7	C	8.00	16.84	288.00	-	6.40	121.60	9.60	1.34	1.34
8	C	8.00	12.62	256.00	4.27	-	81.07	6.40	0.90	0.90
9	C	8.00	8.42	125.00	2.61	1.54	78.79	6.22	0.87	0.87

**Table 5:** Materials of single-factor test required for microcapsules preparation.

Sample (#)	Urea Batch	Urea (g)	Formaldehyde (g)	Distilled Water for Wall Material (mL)	Gum Arabic (g)	Span-80 (g)	Triton X-100 (g)	Distilled Water for Core Material (mL)	Core Materials (g)	NaCl (g)	SiO_2_ (g)
10	A	8.00	8.42	125.00	1.27	1.49	-	52.52	4.15	0.58	0.58
11	A	8.00	8.42	125.00	2.01	0.75	-	52.52	4.15	0.58	0.58
12	A	8.00	8.42	125.00	2.76	-	-	52.52	4.15	0.58	0.58
13	A	8.00	8.42	125.00	2.25	-	0.51	52.52	4.15	0.58	0.58
14	A	8.00	8.42	125.00	1.74	-	1.02	52.52	4.15	0.58	0.58

**Table 6:** Materials table of blended UV primer.

Addition Rate (%)	Mass of the Thermochromic Microcapsules (g)	Mass of the UV Primer (g)
0	0.000	1.000
5	0.050	0.950
10	0.100	0.900
15	0.150	0.850
20	0.200	0.800
25	0.250	0.750
30	0.300	0.700

**Table 7:** Analysis of the yield rate of microcapsules by the orthogonal test.

Sample (#)	Urea Batch	W_F_:W_U_	HLB Values	Core–Wall Ratio	Yield (%)
1	A	1.6:1	8.00	1:1	41.10
2	A	1.2:1	10.00	1:1.5	34.03
3	A	0.8:1	13.40	1:2	43.03
4	B	1.6:1	10.00	1:2	21.70
5	B	1.2:1	13.40	1:1	25.14
6	B	0.8:1	8.00	1:1.5	44.54
7	C	1.6:1	13.40	1:1.5	24.60
8	C	1.2:1	8.00	1:2	29.05
9	C	0.8:1	10.00	1:1	41.26
k1	39.39	29.13	38.23	35.83	
k2	30.46	29.41	32.33	34.39	
k3	31.63	42.94	30.92	31.26	
R	8.93	13.81	7.31	4.57	
Level	A > D > B > C				
Best Level	A1	B3	C1	D1	
Best process	A1B3C1D1				

**Table 8:** Analysis of the results of an orthogonal test of microcapsule encapsulation rate.

Sample (#)	Urea Batch	W_F_:W_U_	HLB Values	Core–Wall Ratio	Encapsulation Rate (%)
1	A	1.6:1	8.00	1:1	71.0
2	A	1.2:1	10.00	1:1.5	57.0
3	A	0.8:1	13.40	1:2	27.0
4	B	1.6:1	10.00	1:2	42.0
5	B	1.2:1	13.40	1:1	43.0
6	B	0.8:1	8.00	1:1.5	47.0
7	C	1.6:1	13.40	1:1.5	50.0
8	C	1.2:1	8.00	1:2	49.0
9	C	0.8:1	10.00	1:1	42.0
k1	51.7	54.3	55.7	52.0	
k2	44.0	49.7	47.0	51.3	
k3	47.0	38.6	40.0	39.3	
R	7.7	15.7	15.7	12.7	
Level	B = C > D > A				
Best Level	A1	B1	C1	D1	
Best process	A1B1C1D1				

**Table 9:** The results of the orthogonal test of microcapsule Δ*E* and formaldehyde emissions.

Sample (#)	Low-Temperature Color Difference Value (−20 °C)			High-Temperature Color Difference Value (60 °C)			Δ*E*	Formaldehyde Emission (mg/m^3^)
	*L*_1_	*a*_1_	*b*_1_	*L*_2_	*a*_2_	*b*_2_		
1	68.156	1.994	13.206	69.400	1.975	15.175	2.329	1.488
2	70.219	0.863	4.506	71.100	0.225	5.050	1.216	1.397
3	63.031	−0.381	−16.675	63.900	−2.050	−16.425	1.898	1.488
4	69.500	−0.188	3.850	70.450	−0.900	4.550	1.378	1.463
5	65.338	1.838	−5.156	66.525	1.000	−4.925	1.471	1.364
6	73.225	−0.994	−10.000	74.475	−1.500	−7.825	2.559	1.299
7	65.450	−1.381	−12.344	66.600	−2.700	−12.000	1.783	1.479
8	67.544	0.413	−12.100	69.925	−1.225	−10.125	3.500	1.397
9	74.675	2.106	−1.075	76.100	1.100	−0.275	1.919	1.463

**Table 10:** Analysis of the results of the orthogonal test of microcapsule ΔE.

Sample (#)	Urea Batch	W_F_:W_U_	HLB Values	Core–Wall Ratio	Δ*E*
1	A	1.6:1	8.00	1:1	2.329
2	A	1.2:1	10.00	1:1.5	1.216
3	A	0.8:1	13.40	1:2	1.898
4	B	1.6:1	10.00	1:2	1.378
5	B	1.2:1	13.40	1:1	1.471
6	B	0.8:1	8.00	1:1.5	2.559
7	C	1.6:1	13.40	1:1.5	1.783
8	C	1.2:1	8.00	1:2	3.500
9	C	0.8:1	10.00	1:1	1.919
k1	1.814	1.830	2.796	1.906	
k2	1.803	2.063	1.504	1.853	
k3	2.401	2.125	1.718	2.259	
R	0.598	0.295	1.292	0.406	
Level	C > A > D > B				
Best Level	A3	B3	C1	D3	
Best process	A3B3C1D3				

**Table 11:** Analysis of the results of the orthogonal test of formaldehyde positive formaldehyde emission processing data.

Sample (#)	Urea Batch	W_F_:W_U_	HLB Values	Core–Wall Ratio	Positive Formaldehyde Emission Processing Data (mg/m^3^)
1	A	1.6:1	8.00	1:1	0.511
2	A	1.2:1	10.00	1:1.5	0.602
3	A	0.8:1	13.40	1:2	0.511
4	B	1.6:1	10.00	1:2	0.536
5	B	1.2:1	13.40	1:1	0.635
6	B	0.8:1	8.00	1:1.5	0.700
7	C	1.6:1	13.40	1:1.5	0.520
8	C	1.2:1	8.00	1:2	0.602
9	C	0.8:1	10.00	1:1	0.536
k1	0.541	0.522	0.604	0.561	
k2	0.624	0.613	0.558	0.607	
k3	0.553	0.582	0.555	0.550	
R	0.083	0.091	0.049	0.057	
Level	B > A > D > C				
Best Level	A2	B2	C1	D2	
Best process	A2B2C1D2				

**Table 12:** Normalization score analysis of results of orthogonal tests.

Sample (#)	*n*				Average of Equal-Weighted Total Scores
	Urea Batch	W_F_:W_U_	HLB Values	Core–Wall Ratio	
1	92.277	100.000	66.539	73.000	82.954
2	76.403	80.282	34.745	86.000	69.357
3	96.610	38.028	54.228	73.000	65.467
4	48.720	59.155	39.373	76.5714	55.955
5	56.444	60.563	42.031	90.714	62.438
6	100.000	66.197	73.110	100.000	84.827
7	55.231	70.422	50.949	74.286	62.722
8	65.222	69.014	100.000	86.000	80.059
9	92.636	59.155	54.823	76.571	70.796

**Table 13:** Factor analysis of the microcapsule scores of orthogonal tests.

Sample (#)	Urea Batch	W_F_:W_U_	HLB Values	Core–Wall Ratio	Average of Equal-Weighted Total Scores
1	A	1.6:1	8.00	1:1	82.954
2	A	1.2:1	10.00	1:1.5	69.357
3	A	0.8:1	13.40	1:2	65.467
4	B	1.6:1	10.00	1:2	55.955
5	B	1.2:1	13.40	1:1	62.438
6	B	0.8:1	8.00	1:1.5	84.827
7	C	1.6:1	13.40	1:1.5	62.722
8	C	1.2:1	8.00	1:2	80.059
9	C	0.8:1	10.00	1:1	70.796
k1	72.592	67.210	82.613	72.063	
k2	67.739	70.618	65.369	72.302	
k3	71.192	73.696	63.542	67.160	
R	4.853	6.486	19.071	5.142	
Level	C > B > D > A				
Best Level	A1	B3	C1	D2	
Best process	A1B3C1D2				

**Table 14:** The results of single-factor tests of the microcapsule.

Sample (#)	Low-Temperature-Color Difference Value (−20 °C)			High-Temperature Color Difference Value (60 °C)			Yield (%)	Encapsulation Rate (%)	Δ*E*	Formaldehyde Emission (mg/m^3^)
	*L*_1_	*a*_1_	*b*_1_	*L*_2_	*a*_2_	*b*_2_				
10	78.8	2.3	−1.425	79.35	5.375	−1.3	41.65	46	3.126	1.405
11	79.325	2.1	−2.45	79.9	5.175	−2.2	49.58	39	3.138	1.364
12	82.15	3.1	−0.725	82.775	1.1	0.025	45.16	42	2.225	1.323
13	86.575	1.2	2.725	86.4	1.925	2.8	46.02	43	0.749	1.340
14	77.925	6.725	−2.25	79.65	2.625	−1.075	43.29	45	4.600	1.310

**Table 15:** Normalization score analysis of results of single-factor tests.

Sample (#)	*n*				Average of Equal-Weighted Total Scores
	Yield (%)	Encapsulation Rate (%)	Δ*E*	Formaldehyde Emission (mg/m^3^)	
10	84.020	100.000	67.953	86.212	84.546
11	100.000	84.783	68.213	92.162	86.290
12	91.087	91.304	48.375	98.113	82.219
13	92.834	93.478	16.293	95.646	74.563
14	87.315	97.826	100.000	100.000	96.285

**Table 16:** Characteristic peaks of the infrared spectrum.

Wave Numbers (cm^−1^)	Characteristic Peak	Substance	Formation Reasons
3321	-OH	Bisphenol A	Telescopic Vibration Peak
2922	C-CH_3_	Bisphenol A	Telescopic vibration peaks
1513 and 1463	Benzene ring C=C	Bisphenol A	Telescopic vibration peak
1251	C-OH	Bisphenol A	Absorption peak
1055	C-O	Bisphenol A	Telescopic vibration peak
1613	Lactone ring carbonyl C=O	Crystal violet lactone	Telescopic vibration peak
1177 and 1055	Ester group C-O-C	Crystal violet lactone	Symmetric telescopic vibration peak
1635	The ester carbonyl of the non-endo ring structure C=O	Core materials	Absorption peak
3321	-OH in the carboxyl group	Core materials	Absorption peak
3353	N-H, O-H	Wall materials	Stretching vibration peak
2963	-CH_2_-	Wall materials	Asymmetric stretching vibration
1566	The N-H of the amide	Wall materials	Bending vibration
1390	C-N	Wall materials	Stretching vibration peak
1136	CH_3_O	Wall materials	Absorption peak

**Table 17:** Effect of adding microcapsule 11# and microcapsule 14# at different amounts on the glossiness and transmittance of paint films.

Sample (#)	Addition Rate (%)	Glossiness (GU)			Transmittance (%)	Losing Glossiness Rate (%)
		20°	60°	85°		
11	0	73.7	112.1	91.6	94.06	-
	5	15.4	46.7	43.5	87.73	58.34
	10	14.3	40.9	47.1	84.35	63.51
	15	8.6	35.8	77.2	81.58	68.06
	20	7.3	28.8	62.1	75.02	74.31
	25	11.4	41.0	57.0	65.31	63.43
14	0	73.7	112.1	91.6	94.06	-
	5	62.3	89.7	74.2	92.43	19.98
	10	62.9	80.3	55.8	88.33	28.37
	15	83.3	88.3	67.4	85.34	21.23
	20	8.6	35.2	58.4	81.90	68.60
	25	10.1	35.9	53.5	78.41	67.98

**Table 18:** Effect of adding microcapsule 11# or microcapsule 14# on the elongation at break and roughness of UV primer.

Sample (#)	Addition Rates (%)	*e* (%)	Roughness (μm)
11	0	10.6	0.089
	5	13.2	0.468
	10	15.3	0.619
	15	12.8	0.196
	20	3.8	0.239
	25	1.8	0.310
14	0	10.6	0.089
	5	16.8	0.212
	10	17.4	0.153
	15	16.3	0.182
	20	6.7	0.201
	25	4.0	0.376

## Data Availability

Data are contained within the article.
